# Genetic Variations in Vesicoureteral Reflux Sequelae

**DOI:** 10.3390/pathogens5010014

**Published:** 2016-02-02

**Authors:** David S. Hains, Andrew L. Schwaderer

**Affiliations:** 1 Innate Immunity Translational Research Center, Children’s Foundation Research Institute, Le Bonheur Children’s Hospital, Memphis, TN 38103, USA; 2 Department of Pediatrics, Nationwide Children’s Hospital, Columbus, OH 43205, USA; andrew.schwaderer@nationwidechildrens.org

**Keywords:** vesicoureteral reflux, genetics, copy number variations, urinary tract infections

## Abstract

Urinary tract infections (UTI) are a common condition in children. Vesicoureteral reflux (VUR) represents a common associated condition with childhood UTI. UTI susceptibility appears to have a genetic component based on family and UTI cohort studies. Targeted analysis of innate immune system genetic variations indicate that these variations are important in UTI susceptibility. In this overview, we discuss how current cohorts and genetic strategies can be implemented to discover new susceptibility loci in patients with UTI.

## 1. Introduction

Under normal circumstances, the urinary tract is a sterile system. In children, 3%–7% of girls and 1%–2% of boys will experience a urinary tract infection (UTI) by age 6 [1]. UTIs, while common, can lead to serious complications if infections ascend into the kidney and cause acute pyelonephritis (APN). These complications include renal scarring, hypertension, and chronic or end-stage kidney disease.

Vesicoureteral reflux (VUR) is one of the most common anomalies associated with APN in children. VUR is the abnormal backflow of urine from the bladder to the kidneys and affects 1%–2% of all children [2,3]. Up to 60% of children with febrile UTI also have VUR [4,5]. A third of children with both UTI and VUR will experience recurrent UTIs. VUR alone does not account for the entire risk of bacteria invading the urinary tract. Only 20% of children with VUR will develop a UTI [6,7,8]. After corrective surgery, children who once had VUR continue to have UTIs, but are less likely to experience febrile UTIs, perhaps because they no longer have an open conduit for bacteria to reach the upper tract [9].

Clinicians have had difficulty determining a consensus for treatment of children with VUR. Current management strategies are focused around preventing UTIs while limiting a child’s exposure to potential side effects from medicines or invasive procedures. Because only a small fraction of children will have recurrent UTI and most will “out-grow” their VUR, the debate regarding how best to approach a child with VUR continues. A child with VUR can be treated with daily antibiotic prophylaxis, have corrective procedures performed, or be carefully watched and intervention initiated when recurrent UTIs occur. Our research group studies why only certain children with VUR get UTIs and why only some develop sequelae. This review of the presentation will serve as an overview for why studies in the genetics of UTI are critical for directing medical decision making in patients at risk of UTI complications.

## 2. Heritability of UTI Susceptibility

A growing body of literature exists implicating the genetic contribution to UTI susceptibility. Several studies have identified inheritance patterns that confer higher UTI risk in family members of affected individuals [10,11,12,13,14]. Interestingly, the type of UTI (cystitis *versus* APN) can also be seen to be over-represented in different families [10,11]. Genetic UTI risk does not appear to follow Mendelian inheritance patterns such as dominant *versus* recessive but rather appears to be a complex genetic trait. Therefore, UTI susceptibility is likely a polygenic phenomenon with environmental and host factors also playing a role.

## 3. The Innate Defense of the Kidney and Urinary Tract

The host innate immune system is the first-line defense of the kidney and urinary tract to prevent microbial invasion. The innate immune system, unlike the adaptive immune system, has constitutively active components and components that are rapidly up-regulated upon microbial presence. The host’s innate immune system is complex but simplistically consists of antimicrobial peptides and molecules that rapidly kill bacteria, chemokines and cytokines that promote an inflammatory response, pattern recognition receptors that recognize pathogens, and phagocytes that consume bacteria [15,16]. These components work in concert to rapidly destroy invading pathogens and activate the inflammatory response. Genetic variations in all these components have been implicated in UTIs or other inflammatory/infectious diseases.

## 4. Genetic Variations in Innate Immunity Genes

Genetic variations in humans range from single nucleotide polymorphisms (SNPs) to large microscopically visible chromosome defects. SNPs occur once every 300 base pairs across the genome and represent a common source of genetic variation [17]. On the other hand, duplications, deletions, or insertions of 1000 base pairs or larger have been identified in humans and designated DNA copy number variations (CNVs). Approximately 0.4% of the genomes of unrelated people differ with respect to DNA copy number variations (CNVs). These changes in DNA copy number can have profound effects on gene dosage, and alterations in DNA copy number play a key role in disease susceptibility and resistance [18]. For example, many genes exist as multiallelic genes and diverge from the conventional diploid copy number that conventional knowledge has taught us. For example, humans possess 1–12 copies of *DEFB4*, with four copies occurring most frequently. CNVs in the *DEFB4* gene and other key components of innate immunity have been reported in susceptibility to Crohn’s disease and HIV [19,20,21,22]. Thus, CNVs in innate immunity genes that are critical to the innate defense of the kidney and urinary tract could lead to UTI susceptibility.

## 5. Designing an Genomic Approach to Discover UTI Risk Alleles

The first challenge in an unbiased approach for determining genetic risk factors that contribute to UTI risk is recruiting a well-phenotyped, longitudinal cohort of UTI-susceptible individuals. The Randomized Intervention for Children with Vesicoureteral Reflux (RIVUR) Study (ClinicalTrials.gov Identifier NCT00405704) recently completed in North America [23]. All children in the RIVUR Study had documented VUR by voiding cystourethrogram, were 2 months to 6 years of age, and had at least one but no more than two documented UTIs at the beginning of the study. Children participating in the RIVUR Study were randomly assigned to one of two treatment groups. One group received antibiotic prophylaxis, while the control group received a placebo. All children in the RIVUR study were followed in the study for 2 years and monitored for UTIs. DNA was also collected on children participating in the study. Very few studies/cohorts exist that are as well characterized as the RIVUR study in pediatrics.

The second challenge to determine what genetic determinants may contribute to UTI risk in humans is selecting the appropriate genome-wide approach to assess genetic variations. Classic approaches to determine genetic causality rely on either rare variants to be over-represented for rare diseases or large pedigrees with genetic similar individuals with affected and unaffected individuals. Applying these techniques to a complex genetic trait such as VUR and UTI risk represented by the RIVUR study cohort would likely cause key disease modifying genes to be overlooked [24]. Furthermore, many genes that contribute to inflammatory pathways and innate immunity are multi-allelic. For example, many of the alpha and beta defensins as well as the chemokine genes exist as multiple copies [19,21,25]. Presumably, having high or low copies in these genes will give different infectious and inflammatory responses. Assessing copy number differences in multi-allelic genes can be challenging as well [26]. Instead of embarking on a bioinformatic challenge with millions of variations that may be important, one could consider alternative genetic approaches such as array comparative genomic hybridization (aCGH) to assess the contribution of CNVs to UTI susceptibility.

**Figure 1:**
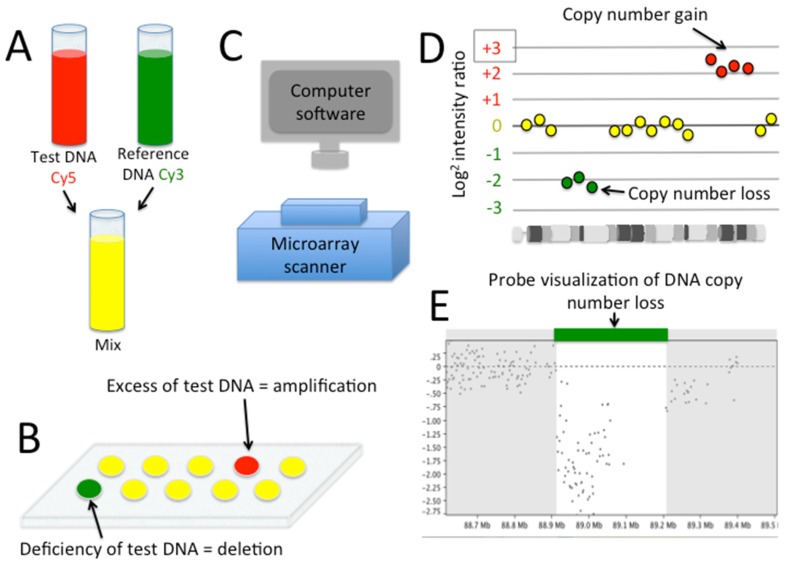
Comparative Genomic Hybridization. (**A**) Equal amounts test and reference DNA samples are labeled respectively and mixed; (**B**) Mixture is applied to an array and allowed to hybridize; (**C**) Red and green signal intensity is detected and mapped to genome location; (**D**) Software clusters high and low intensity probes according to log^2^ intensity ratio of test to reference; (**E**) Example of a number of probes representing a copy number loss in test sample.

High-resolution aCGH utilizes a whole-genome tiling array with oligonucleotide probes (25–85 base pairs) spaced every 1–2 kilobases and covers the entire genome. Patient DNA is labeled with a fluorescent dye and a reference sample is labeled with a different colored fluorophore (Figure 1). Equal amounts of DNA from the patient and reference are mixed and hybridized to the array [27]. Next, the amount patient DNA that hybridizes relative (comparative) to the reference sample is digitally analyzed and mapped across the genome. Probe density and quality determine the level of resolution, but present day CGH platforms allow for detection of single gene variations and even deletions/amplifications as small as 4 base pairs [28]. Furthermore, aCGH data can be compared across multiple patients and controls to look for specific CNVs as well as biological pathways that are perturbed in cohorts of diseases using simple software programs that run on a desktop computer such as Nexus Copy Number (Biodiscovery, El Segundo, CA, USA). Because CNVs are much more rare than SNPs, a typical patient may only have a few hundred CNVs compared to potentially millions of variants produced with next-generation sequencing. Thus, for analyzing gene dosing effects in multi-allelic genes that may result in a spectrum of phenotypes across a population, aCGH provides a cost-effective and bioinformatically more appealing option.

## 6. Conclusions

Variations in innate immunity genes lead to UTI susceptibility. In this review, we provide a novel approach and rationale for using aCGH to interrogate genomes for multi-allelic genes such as the alpha and beta-defensin loci that may be critical in UTI susceptibility.
